# Dental Exhibits

**Published:** 1889-04

**Authors:** 


### DENTAL EXHIBITS.

The Exhibition of Novelties in our dental meetings is one of the most interesting features of the session. The relation between practitioner and dealer is a mutually dependent one, the former depending upon the latter for his supplies, and the dealer upon the consumer for the disposal of his goods. The exhibit therefore forms a valuable annex to our dental conventions, enabling the dentist to see, and the dealer to bring at once before a large number of the profession the latest novelties in use. Each year adds its proportion to the already long list of useful appliances, and the late exhibits in their entirety show that the mechanical and labor-saving workers of the profession are not far behind the great advance being made in the science itself.

